# Sexual and reproductive health among adolescents in vulnerable contexts in Mexico: Needs, knowledge, and rights

**DOI:** 10.1371/journal.pgph.0002396

**Published:** 2023-11-01

**Authors:** Aremis Villalobos, Fátima Estrada, Celia Hubert, Leticia Torres-Ibarra, Alejandro Rodríguez, Irma Romero, Raffaela Schiavon, Lourdes Campero

**Affiliations:** 1 Center for Population Health Research, National Institute of Public Health, Cuernavaca Morelos, Mexico; 2 CONACYT-INSP, Cuernavaca Morelos, Mexico; 3 Independent Consultant, Mexico City, Mexico; UNAM: Universidad Nacional Autonoma de Mexico, MEXICO

## Abstract

Ensuring sexual and reproductive health, and rights for adolescents entails the prevention of early pregnancies, which are widely recognized as a public health problem. Based on the ecological model for early pregnancy, this article identifies the healthcare requirements for preventing unintended adolescent pregnancies in predominantly indigenous communities in Chiapas, Mexico. Using a convergent parallel mixed-methods study design, we surveyed adolescents (12–15 years old) and health personnel, organized focus groups with adolescents and their parents, and conducted in-depth interviews at the individual, family, school and community levels. Results showed that adolescents recognized their right to receive sexuality education (64.5%) as well as information on contraceptive methods (53.0%), with indigenous language speakers and individuals living in overcrowded households less likely to know about these rights. Parents of adolescents knew little about contraception and pregnancy. School teachers lacked necessary tools for offering comprehensive sexuality education. A traditional, patriarchal perspective predominated among participants, fostering gender inequalities. In conclusion, it is essential to implement multifocal strategies under a human-rights, intercultural, and health-equity approach. Special attention should be directed to the spheres in which adolescents interact, and efforts should focus on improving knowledge, empowering adolescents, and enhancing their access to sexual and reproductive health resources.

## Introduction

The importance of sexual and reproductive health (SRH) in adolescents has been recognized worldwide and was incorporated into the 2030 United Nations agenda in the form of two specific goals: universal access to family planning services and access to sexuality education [1]. Ensuring Adolescent Sexual and Reproductive Health and Rights (ASRHRs) entails the prevention of early pregnancies, widely recognized as a public health problem [2–4]. With an adolescent fertility rate (AFR) of 69.5 births per 1000 women aged 15 to 19 years in 2019, Mexico ranks first among Organization for Economic Cooperation and Development countries as in adolescent pregnancies [5]. Adolescent pregnancy negatively affects health, school attendance, present and future income, access to employment opportunities, and human development. It is more prevalent among the population with disadvantaged characteristics such as rural residence, indigenous and Afro-Mexican communities, and low socioeconomic status. In these contexts, traditional gender roles prevail, which limit the autonomy and exercise of the rights of adolescents, particularly those of women [6].

Providing comprehensive sexuality education (CSE) together with quality and friendly SRH services, both recognized as fundamental rights, is intimately intertwined with the empowerment of adolescents and the prevention of unintended pregnancies [7]. However, studies have shown that CSE contents have yet to be provided in an integrated, homogeneous, and continuous manner, hindering the teaching of all the topics proposed in national and international recommendations [8]. Additionally, evidence exists that adolescent SRH services are inadequate and insufficient [9–12], particularly among the most vulnerable populations [13]. For instance, indigenous women experience dramatic SRH inequities [14–16] as well as barriers to information and restricted access to contraceptive methods [16–18].

Inequality in adolescent fertility rates across different population groups is especially noticeable in Mexico. In locations with lower socioeconomic status, particularly with a higher proportion of indigenous population, fertility rates are greater. The indigenous population’s AFR in 2020 was 87.1 births per 1000 women, as opposed to 69 births for non-indigenous women. The state of Chiapas, the one with the highest proportion of indigenous residents (28% vs. 7% nationally) had the second-highest adolescent AFR on a national level (84.9 births per 1000 women ages 15–19) [6]. Although in Mexico, the importance of a comprehensive sexual education has been recognized for empowering adolescents, it is important to note that that there is no one strategy that will work for all, especially in a country with high social inequalities. Evidence about SRH needs are scarce in rural and indigenous population where the need is greatest [6].

To achieve a more in-depth understanding of the problems affecting ASRHRs, it is essential to address the various social spheres in which adolescents interact. Safe, as opposed to high-risk behaviors are shaped at the family, school and community levels [19].

We aimed to identify the healthcare requirements that would support efforts to prevent adolescent pregnancy in two predominantly indigenous communities in Chiapas, Mexico. Our study explored the levels of knowledge among adolescents concerning contraceptive methods and sexual and reproductive rights. In addition, to gain a more comprehensive understanding of the behaviors and dynamics of this context we explore the perceptions of adolescents, mothers and fathers of adolescents, schoolteachers, health personnel, young women with a history of adolescent pregnancy, and community leaders in relation to the SRH needs.

## Materials and methods

### Ethics statement

The study was conducted according to the guidelines of the Declaration of Helsinki. The protocol, instruments and informed consent forms were approved by the Research Ethics Committee of the National Institute of Public Health, in Mexico (CI: 1692). Written informed consent was obtained from all parents or guardians of the adolescents, aged <17 years, who participated in the study; the adolescents read the consent document prior to participating in the study.

### Study design

**Fig 1:**
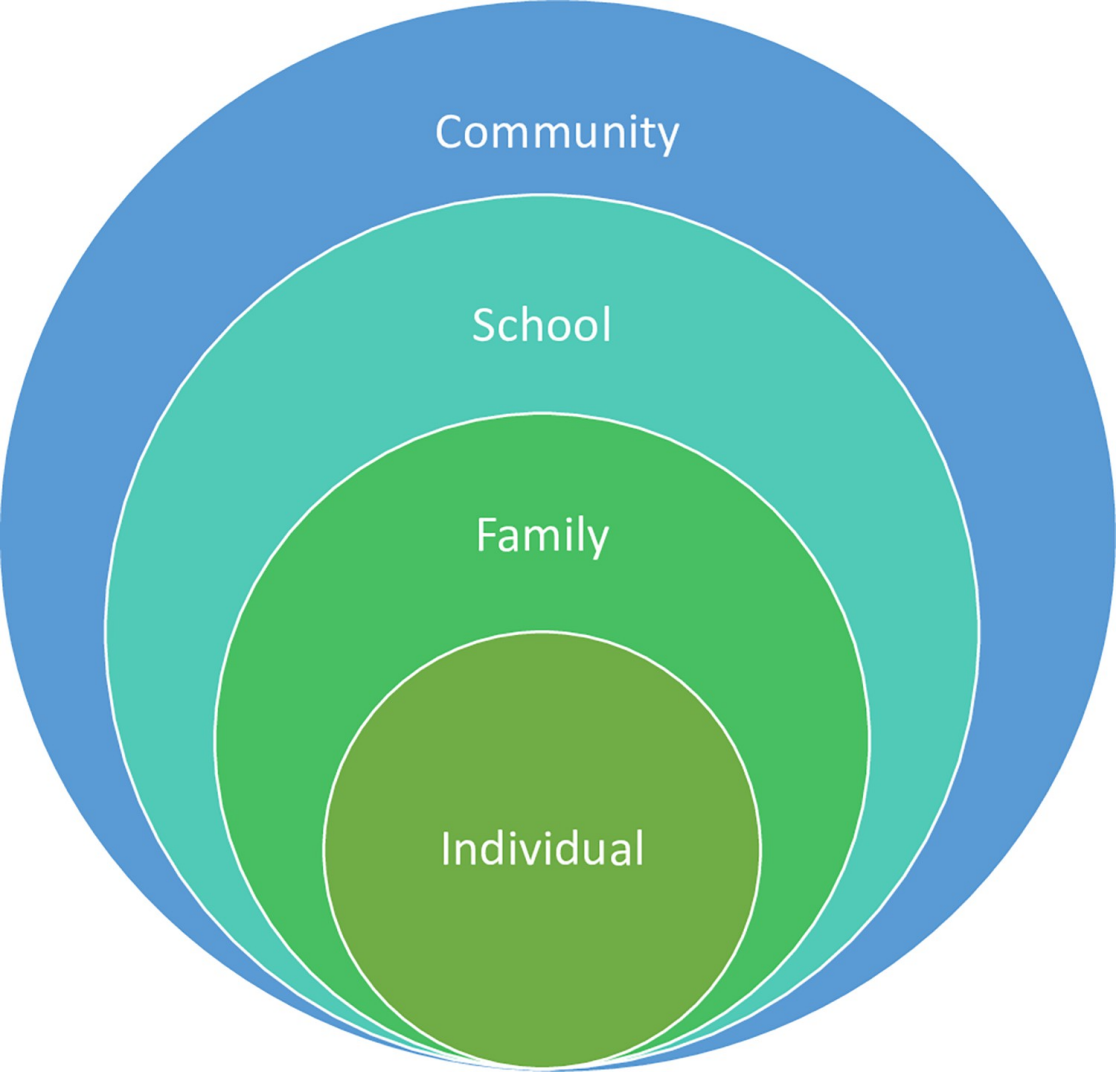
Ecological model for early pregnancy.

A convergent parallel mixed-methods design was used to collect and interpret quantitative and qualitative information [20, 21]. Equal weight was assigned to the components of both methods. First, we analyzed the two components independently. Then, to understand the ASRHR challenges in preventing early pregnancies in vulnerable contexts, we analyzed quantitative and qualitative information following the perspective of the socio-ecological model for early pregnancy (Fig 1). This model is a theory-based framework for understanding and exploring how social determinants across different levels, beyond the individual, influence health outcomes. This model is widely accepted for informing adolescent pregnancy prevention programs that recognize that adolescents’ behaviors are influenced by their peers, family, organizations to which they belong and the communities in which they live [22, 23].

### Setting

Between March 2021 and April 2022, we undertook the study in Chiapas, the state with the highest proportion of population living in poverty (67.4% vs. 36.3% nationally in 2022) [24] and the highest proportion of indigenous residents (28% vs. 7% nationally) [25]. We selected an urban and a rural community near the state capital, both located in the highlands and inhabited by a large percentage of people from the Tseltal and Tsotsil ethnic groups were selected [26]. This part of Chiapas is well known for its large number of national and international migrants. Tourism is the main economic activity in the urban community, while the production and sale of handicrafts and flowers are predominant among both populations.

We selected five public lower secondary schools serving a large number of adolescent students in the area and belonging to the most marginalized sectors in both communities. For instance, the selected schools in San Cristobal attend about 30% of the total of lower secondary students in this municipality [27]. Although the reference age group for attending lower secondary is 12 to 14 years, there are students who start this level at age 11 and students who complete it after age 14. Since this is an observational, exploratory study, we did not perform sample size calculations. Once we selected the communities, we chose the schools with the largest number of students, assuming that this would allow us to have a greater variability in our population.

### Participants

#### Quantitative component

We included adolescents attending second and third grade in the selected lower secondaries. These students were 12 to 15 years of age. They completed a self-administered questionnaire (S1 Text). Participants included medical, nursing, psychology, and social-work staff as well as community-level promoters involved in adolescent SRH activities. Those providing primary- and secondary-healthcare services to adolescents completed an online, self-administered survey. In order to reduce social desirability bias and ensure privacy, we used an audio-computer assisted self- interview (ACASI) method for the adolescents’ questionnaires and Google Forms for the healthcare providers online survey (S1 Text).

#### Qualitative component

This facet of the study featured 73 semi-structured interviews with academic and health personnel, community leaders, and young women with a history of adolescent pregnancy. Twenty-three focus groups sessions were organized with adolescents ‘students, parents of adolescents, and 40 “mystery clients” visits to healthcare services to assume the role of adolescent users in order to obtain a realistic picture of local adolescent. We used information obtained in the interviews, and focus groups to create the mystery clients’ guides, and cases.

**Fig 2:**
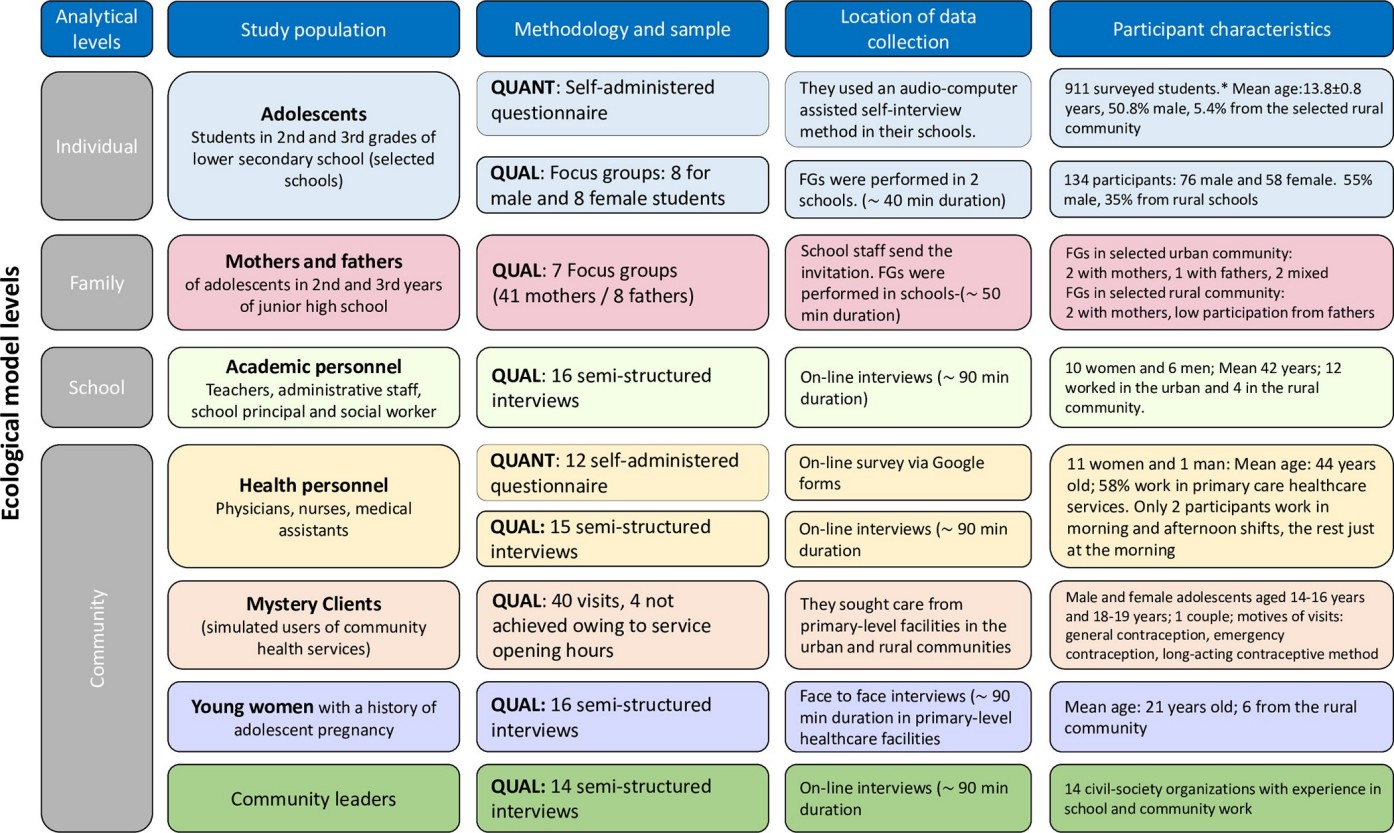
Study populations, methods, sample sizes, location of data collection, and participant characteristics by level of analysis. *Although all students were invited simultaneously, data collection for the quantitative and qualitative components took place on different days; therefore, the same students did not necessarily participate in both components. QUANT = quantitative; QUAL = qualitative; FG = focus groups.

Fig 2 describes the analytical levels, study population and overall participant characteristics in more detail. The general themes explored during the quantitative component pertained to the knowledge and experiences of participants regarding sexual and reproductive health. They also concerned the perceptions of respondents on adolescent pregnancy prevention. Regarding the health personnel and mystery clients specifically, data were gathered on care provided at local health services. They used a standardized script to simulate real-life queries, observe the performance of personnel and/or elicit information related to four of the five World Health Organization criteria for “adolescent-friendly services” [4]. Each visit was followed by debriefing and recording of details on a standardized form.

### Data analysis and integration

The quantitative component included a descriptive analysis of the information gathered from the adolescents and health personnel. For the adolescent group, we analyzed whether they were aware of their sexual and reproductive rights and had knowledge of contraceptive methods; specifically, the intrauterine device (IUD), condom, implant, and contraceptive pills. For this purpose, we constructed two dichotomous variables in order to identify the most knowledgeable adolescents, that is, those with the greatest amount of information on their sexual and reproductive rights (above average: >3 rights) and on contraceptive methods (above average: >2 contraceptive methods).

We adjusted a multivariate logistic-regression model to explore the association of each sociodemographic variable with knowledge regarding sexual and reproductive rights and contraceptive methods. Data analysis was performed using Stata/MP 17.0. (S1 Data).

For the qualitative component, we transcribed and systematized the recordings of the interviews and focus group sessions. We ensured data variability by incorporating the perspectives of diverse social actors, collecting information from different sites, and integrating a variety of topics in the objective. The analytical categories emerged as the information was gathered and analyzed from the qualitative data. For the coding process, we worked in triads, constantly verifying and discussing the preliminary findings derived from the information. The analytical codes and categories were assigned by consensus [28].

Finally, based on the qualitative and quantitative information, we conducted a deductive exercise based on the socio-ecological model, in which we identified cross-cutting factors, as well as specific ones by level and informant. This allows showing a general overview of adolescent pregnancy among predominantly indigenous communities in Chiapas, Mexico.

## Results

Fig 2 presents the characteristics of the participating adolescents, mothers and fathers of adolescents, schoolteachers, health personnel, young women with a history of adolescent pregnancy, and community leaders who participated in the study. A total of 911 adolescents of mean (standard deviation [SD]) age 13.8 (0.8) years were surveyed; of these, 50.8% were male, 24.0% spoke an indigenous language, 5.4% resided in rural areas and 12.6% lived in overcrowded households (more than 2.5 people per room). In total, 12 health personnel of mean (SD) age 44 (6.7) were surveyed; of which, 91.7% were female and 58.3% worked in health units (Table 1).

**Table 1:** Characteristics of adolescents and health personnel.

	Interviewees
**Students**	
**Total**	911
**Mean age (SD)**	13.8 (0.8)
**Sex**	
Female	448 (49.2)
Male	463 (50.8)
**Is an indigenous-language speaker**	
Yes	219 (24.0)
No	692 (75.9)
**Lives in a rural community**	
Yes	49 (5.4)
No	862 (94.6)
**Lives in an overcrowded household**	
Yes	115 (12.6)
No	796 (87.4)
**Health personnel**	
Total	12
**Mean age (SD)**	44 (6.7)
**Sex**	
Female	11 (91.7)
Male	1 (8.3)
**Characteristics of the type of health facility**	
Health unit	7 (58.3)
Hospital	5 (41.7)

All values are n (%) unless stated

SD, standard deviation

Following the socio-ecological perspective, we present the information by level identifying transversal factors across them. We first present the results by level, and then the factors that converge in more than one level.

### Factors at the individual-level

Adolescents lack the knowledge and skills to exercise their sexual and reproductive health and rights freely, fully and in a healthy way. According to their self-reports, adolescents were aware of three sexual and reproductive rights on average, the most common of which were the rights to receive sexuality education (64.5%) and obtain information on contraceptive methods (53.0%) and receive contraceptive methods at drugstores and/or healthcare facilities unaccompanied by an adult (19.9%). In contrast, few were aware of their right to enjoy a satisfying sexual life (5.4%), have sex when desired (12.9%), live with a partner (18.2%) and obtain contraceptive methods without being accompanied by an adult (19.9%). Female adolescents are unaware of the fact that having rights and opportunities would boost their personal development beyond motherhood.

Adolescents are reluctant to go to healthcare facilities because they perceive a lack of discretion and confidentiality, and they are embarrassed to request information or contraceptive. Regarding knowledge on contraception, adolescents were acquainted with an average of three contraceptive methods, with most respondents having heard of male condoms (95.6%), contraceptive pills (86.4%), and implants (58.6%). However, only 54.4% and 31.3% knew where they could obtain condoms and contraceptive pills, respectively. The IUD was the least known contraceptive (42.7%), and only 17.7% of those acquainted with this method knew where it could be obtained (Table 2).

**Table 2:** Percentages of adolescents according to their knowledge on sexual and reproductive rights, contraceptive methods and where to find them, and use of sexual and reproductive health services.

	N = 911
**Adolescent Sexual and reproductive rights**	
Average number of rights identified (mean, SD)	3.0 (2.99)
Right to receive sexuality education	588 (64.5)
Right to receive information on contraceptive methods	483 (53.0)
Right to receive contraceptive methods at drugstores and/or healthcare facilities unaccompanied by an adult	181 (19.9)
Right to have sexual relations when desired	118 (12.9)
Right to have a satisfying sexual life	49 (5.4)
Right to decide when to have children	241 (26.5)
Right to decide when to marry or live in union	166 (18.2)
Right to decide who to have children with	193 (21.2)
Right to make decisions freely about one’s body and sexuality	257 (28.2)
Right to have one’s intimacy and private life respected	245 (26.9)
Right to live free from sexual abuse	221 (24.3)
**Knowledge on contraceptive methods**	
Average number of contraceptive methods identified (mean, SD)	2.8 (1.0)
Contraceptive pills	787 (86.4)
Knows where to obtain them	246 (31.3)
Sources of information about this method	
School	624 (79.3)
Home	182 (23.1)
Health personnel	125 (15.9)
Other*	192 (24.4)
IUD	389 (42.7)
Knows where to obtain it	69 (17.7)
Sources of information about this method:	
School	306 (78.7)
Home	95 (24.4)
Health personnel	64 (16.5)
Other*	82 (21.1)
Implant	534 (58.6)
Knows where to obtain it	142 (26.6)
Sources of information about this method	
School	352 (65.9)
Home	147 (27.5)
Health personnel	111 (20.8)
Other*	125 (23.4)
Male condom	871 (95.6)
Knows where to obtain it	474 (54.4)
Sources of information about this method	
School	708 (81.3)
Home	239 (27.4)
Health personnel	170 (19.5)
Other*	231 (26.5)
**Have you ever gone to a health facility or hospital to obtain information or services for contraception, pregnancy or sexually transmitted infections?**	
Yes	93 (10.2)
No	818 (89.8)
**Have you ever thought of going to a health facility to**	
Obtain information on contraceptive methods?	347 (39.6)
Obtain a contraceptive method?	67 (7.6)
Check the contraceptive method you have been using?	29 (3.3)
Have you ever thought of going to a health facility?	500 (57.0)
**Have you talked with your parents about**	
Sex?	
Yes	595 (65.3)
No	316 (34.7)
How to use a condom?	
Yes	403 (44.2)
No	508 (55.8)
How to prevent pregnancy?	
Yes	624 (68.5)
No	287 (31.5)

All values are n (%) unless stated

*Other: the internet, boyfriend/girlfriend or intimate partner, friends and others. IUD, intrauterine device; SD, standard deviation

Being a woman increased the likelihood of being familiar with a larger number of contraceptive methods by 50% (odds ratio [OR] = 1.5; confidence interval [CI]: 1.12, 2.00), and residing in an urban area increased the likelihood 2.6 times (OR = 2.56; CI: 1.33, 4.95). In contrast, speaking an indigenous language and living in an overcrowded household reduced the likelihood of knowing more contraceptive methods by 55% and 33%, respectively, and of enumerating a larger number of sexual and reproductive rights (OR = 0.35 and OR = 0.49, respectively) (Table 3).

**Table 3:** Logistic regression models with the following dependent variables: Increased knowledge of contraceptive methods and increased knowledge of sexual and reproductive rights.

	Contraceptive methods				Sexual and reproductive rights			
	% above average*	Unadjusted OR (CI 95%)	Adjusted OR (CI 95%)	P-value	% above average^#^	Unadjusted OR (CI 95%)	Adjusted OR (CI 95%)	P-value
**Sex**								
Male	61.9	1.00	1.00		28.7	1.00	1.00	
Female	71.2	1.51 (1.14,2.00)	1.50 (1.12, 2.00)	0.006	34.8	1.32 (1.00,1.75)	1.33 (1.00, 1.77)	0.055
**Age, years**								
12–14	67.2	1.00	1.00	0.602	32.3	1.00	1.00	
15–19	63.5	0.85 (0.59,1.21)	0.91 (0.63, 1.31)		28.9	0.85 (0.58,1.24)	0.90 (0.61, 1.32)	0.58
**Speaks an indigenous language**								
No	72.1	1.00	1.00		36.6	1.00	1.00	
Yes	48.9	0.36 (0.27,0.50)	0.45 (0.32, 0.63)	0.00	16.4	0.34 (0.23,0.50)	0.35 (0.24, 0.52)	0.00
**Lives in an urban community**								
No	32.7	1.00	1.00		0.0	-	-	-
Yes	68.5	4.47 (2.42,8.26)	2.56 (1.33, 4.95)	0.005	33.5			
**Lives in an overcrowded household**								
No	67.7	1.00	1.00		33.5	1.00	1.00	
Yes	58.3	0.66 (0.44,0.99)	0.67 (0.44, 1.01)	0.053	19.1	0.46 (0.28,0.76)	0.49 (0.30, 0.80)	0.00

CI, confidence interval; OR, odds ratio

*Knowledge on contraceptive methods is above average when the adolescent know more than 2 contraceptive methods; ^#^Knowledge on sexual and reproductive rights is above average when the adolescent know more than 3 rights

In general, adolescents reported having general knowledge on sexuality, but, as stated by a male rural student in a focus group, they had "little knowledge on sexual relations", many are uninformed about the ways in which pregnancy occurs.

Also, the adolescents mention that they seldom spoke with their parents about SRH and other relevant issues. During a focus group session, a rural female student explained that "they might think I’m asking because I’m pregnant or I’m interested in the [contraceptive] methods because I’m already having sexual relations." Students who did inquire about sexuality, did so indirectly, knowing that their parents also had received limited information when they were young. During an interview at an urban health facility, a young woman who had experienced pregnancy during her adolescence stated the following:

*"Before, parents didn’t talk about sex*. *We thought babies came on a plane! That’s how our parents fooled us, that’s what they used to tell us*… *I only learned about these things when I came [to live] with my husband; my aunt advised me to agree to have sex with him and other things like that"*

Female-student rarely researched this type of information online because they were embarrassed: "I’d feel ashamed if someone saw me looking for this information," said an urban female student at a focus group session. In spite of school was the primary source of information on sexuality and contraception, students rarely asked their teachers about sexuality: "We don’t ask them personal questions," commented one female student from the urban community during a focus group session. Teachers are also embarrassed to talk about these issues in the classroom. They assured us that few teachers addressed the subject of sexuality openly and clearly in the classroom, and those who did, had difficulty eliciting a dialogue.

### Factors at the family-level

Among the adolescents interviewed, 34.7% had addressed the issue of sexuality with their mothers or fathers; 31.5% had never discussed how to avoid pregnancy, and 55.8% had never spoken about how to use condoms (Table 2).

Families have a strongly rooted belief idea that adolescents should replicate their parents’ cultural and reproductive models, and transfer their beliefs, myths and taboos from one generation to the next. “It is affirmed that if grandmothers and mothers have numerous offspring at an early age, then there’s no need to have fewer children. The idea has persisted that a large family is, like, a more powerful family, a more respectable family.”

Furthermore, a lack of information existed among the mothers and fathers of adolescents regarding contraceptive methods and the ways in which pregnancy occurs. These topics were rarely discussed within the couple and even less so with the kids. During a focus group session, the father of an urban student asserted, "I do talk to my children about health, but not about sex, because they’re still young. Maybe they don’t know what it is yet; although, actually, it would be good to talk and tell them what it is."

Several mothers and fathers stated that, besides feeling unsure about the information that should be transmitted, they were embarrassed to talk about sex. They also indicated that their children disliked discussing those topics with them. The following information was offered at a focus group session by the mother of an urban student: "I don’t talk to him about it because he feels very embarrassed. So, I prefer to wait until he asks me or tells me he wants to talk. That’s why I haven’t talked to him about it."

In general, parents felt that, if these issues were to be discussed with their children at all, fathers should talk with their male and mothers with their female children. This was clearly expressed at a focus group session by the mother of an urban student: "I tell my husband, you should talk more with him, he’s a boy.’ I say, ’maybe he’s embarrassed to talk to me, but not to you.‴ And at the same time, parents are reluctant to have their children receive information on matters related to sexuality.

Adults in positions of authority within the family frequently resorted to coercion to prevent adolescents from making informed decisions about their sexual and reproductive lives. Once adolescents formed a couple, often under family pressure, they were pushed to have a child soon as a way of consolidating the marital relationship. A young woman who had experienced an adolescent pregnancy asserted the following at an urban healthcare facility: "We had been together for eight months and I didn’t get pregnant. He wanted to have his baby… My mother-in-law went to buy me an injection and, after a month, I got pregnant."

Domestic violence was a recurrent and internalized fact of life in both communities. Because women were unaware of their rights, very few took ownership.–. Additionally, they did not always view abuse as something negative or inadmissible. One female member of the health personnel at an urban health facility recounted the following:

*“Violence has become so normalized that they say, ‘yes, he’s jealous, but that’s normal,’ or ‘he controls my time, but that’s normal.’ They don’t see it is violence. And when I explain it to them and try to give them all the information, that’s when they say, ’yes, it’s true, that’s not right, but I can’t do anything about it right now. I can’t because I don’t have a support network. I don’t have a way of supporting myself. I don’t work, I’m a housewife. In the community, they won’t let me separate from my husband. I’m very young, I can’t leave him. He’s my livelihood [only source of income]. It would be worse for me.’ These are very complicated situations. The issue of violence is difficult here in Chiapas.”*

### Factors at the school-level

According to the adolescents themselves, school was their most common source of information on contraceptive methods: this was related by 79.3% of those who had heard about contraceptive pills and 81.3% of those who had heard about condoms (Table 2).

The academic personnel, however, lacked the necessary CSE skills. In addition, many were unaware of the advantages of adolescents knowing and taking ownership of their sexual and reproductive rights in order to exercise a healthy, free and satisfying sexual life. One female teacher from the rural community acknowledged her limitations on this subject during an interview:

“*I don’t feel really prepared and I don’t know what I should tell them*. *I’d like to have more information about sexual and reproductive rights*, *sexual relations*, *sexual pleasure*, *sexual diversity*, *negotiation*, *and consent*. *These would be very interesting topics for me*.*”*

The school setting did not encourage open discussion about sex, among other reasons because teachers wanted to avoid having problems with parents for discussing these issues, and because they believe that, in early adolescence, students lack the interest and maturity to comprehend CSE topics. During an interview, one female teacher from an urban school cited the “[…] fear of being punished by the parents… They are minors… How do you give them access to condoms when the community is going to blame us for encouraging ‘my daughter’ or ‘my son’ to have sexual intercourse?”

Nonetheless, some parents agreed that information on contraceptive methods should be disseminated at school. The mother of a rural student expressed the following opinion at a focus group session: “With regard to methods, I’m not very familiar with them, but I’ve heard about some. They’ve given us information here at school, and I think that idea is a good one.”

Sexuality education information was limited, since many adults felt that providing it hastened the initiation of sexual life. One male teacher from an urban school recounted this during an interview:

*“From the experience we’ve had at school, sometimes, this type of conversation or guidance is misunderstood by the authorities, both educational staff and parents. There are parents who think that [we] teachers are giving bad advice to the students, when it is the students who approach us to ask questions.”*

Also, there is a lack of teaching material to work on CSE issues, and poorly trained teachers have difficulty addressing CSE issues, and therefore tend to transmit information according to their own values rather than scientific evidence.

Respondents emphasized potential risks more than the free exercise of their rights. One female teacher from the urban community made the following comment during an interview: "It depends on the teacher, on whether he’s more open, using clear language or [on the contrary] addressing these issues covertly… Some [teachers] are even sexist."

### Community-level factors

Among adolescents surveyed, 10.2% had sought information from a health facility on available services for contraception, pregnancy or sexually transmitted infections, while 57.0% had never considered going to such a facility (Table 2). Health personnel in the study mentioned that the physical space was available for meeting with adolescents and counseling them on SRH. Two staff members reported that counseling was offered during both morning and evening shifts; nine commented that it was provided to most adolescents regardless of their age; three felt that they lacked the ability to discuss sexual and reproductive rights; and two felt that they lacked the capacity to provide counseling (Table 4). And also, youth have limited access to information through mass media and social networks.

**Table 4:** Characteristics of adolescent health services.

	N = 12
**Shifts during which services are specifically available for adolescents**	
Morning	10 (83.3)
Morning and afternoon	2 (16.7)
The health facility has physical space available for receiving adolescents	12 (100.0)
The health facility provides counseling or guidance on sexual and reproductive health	12 (100.0)
The health facility has space designated exclusively for counseling	10 (83.3)
**Type of counseling**	
Individual	1 (8.3)
Group	2 (16.7)
Both	9 (75.0)
**Age range in which counseling is provided**	
All adolescents regardless of age	9 (75.0)
Adolescents ≥15 years old	3 (25.0)
**Provides adolescents with services whether or not they are accompanied by an adult**	
No	2 (16.7)
Yes	10 (83.3)
**How well do you feel you have been trained to talk with adolescents about sexual and reproductive rights**	
Poorly trained	3 (25.0)
Moderately trained	6 (50.0)
Well trained	3 (25.0)
**Provide counseling**	
Poorly trained	2 (16.7)
Moderately trained	7 (58.3)
Well trained	3 (25.0)
**Prevent or deal with situations of family or partner violence and sexual abuse**	
Poorly trained	5 (41.7)
Moderately trained	5 (41.7)
Well trained	2 (16.7)

All values are n (%) unless stated.

#### Community attitudes and beliefs deterring adolescents from exercising their sexual and reproductive rights

The commentaries of various social actors in the study made it clear that the worldview in both communities analyzed have a strong religious component attached to ancestral forms of social organization which, in turn, are based on customs and conventions agreed on by adult community members. Many of the accepted practices ensuing from this worldview constitute barriers to preventing early pregnancy.

According to the cultural values of the communities analyzed, young women are expected to marry after the onset of menstruation (Table 4). In the words of a female staff member interviewed at an urban health facility: "Their authorities allow agreements between families so that girls can marry as soon as they have a menstrual period."

According to the local cultural beliefs, public displays of affection are prohibited, which in some cases favors and in others forces early unions. The following information was provided by a male teacher during an interview at an urban school:

*“They come to study with us, and [when the adults] see the young couple talking, their parents make a deal and marry them off; or they commit their daughter while she’s young, and when she reaches a certain age, an agreement is made for her to go with him.”*

Besides having no autonomy in decisions regarding the couple of which she was a part, if a girl regret leaving home, she is practically forbidden to return. A male urban teacher stated the following:

*“It’s not like in the city, where if her life doesn’t work out, she comes back. Not here. She left. The girl’s family already gave her away. You already left, so forget it, it’s the life you chose for better or for worse.”*

A traditional, patriarchal way of thinking fosters enormous gender inequalities. Women are prevented from having their capacities recognized, are granted only limited rights, and are prohibited from making decisions about their bodies, sexuality and reproduction; they are thus placed in situations of increased vulnerability. Having introjected traditional gender roles, most women have no expectations other than getting married and having children.

#### Difficulties in providing care for adolescents at SRH services

Most health personnel reported offering adolescents care regardless of their age. Nonetheless, it was highlighted that in some cases, information was withheld from those who were not accompanied by an adult, in contravention of the official technical regulations and guidelines. It was also evident that health personnel perceived themselves as being poorly trained, not only to discuss the subject of sexual and reproductive rights [3] and provide counseling [2], but also to prevent or deal with situations of family and partner violence as well as sexual abuse [5] (Table 4). A female health professional interviewed at a rural health facility described her feelings during counseling:

*“I have a hard time talking about the condom technique. Usually, it’s, like, they [the adolescents] feel embarrassed, and sometimes, me too. Like, they intimidate me all of a sudden. I try to be calm, not to laugh, to make things easy for them by using appropriate words so they understand.”*

In addition, health personnel lack the necessary skills to manage counseling from a youth perspective. The following testimony was provided by a female staff member during an interview at an urban healthcare facility:

*“…adolescents normally arrive [at the facility] with their fathers, so when they come in for consultation, they rarely request the method as such. The doctor gives them all the information, but the adolescent does not request it as such, because he [or she] feels pressured by the father.”*

The services often lack an intercultural vision allowing for an approach more suitable to the population. At a rural focus group session, a male mystery client related the following:

*“The hardest thing is asking for information. Also, the nurses don’t understand our language. If they understood our language, it would be easier to ask… Even when they have a translator, sometimes the translator doesn’t care; she just sits there and translates something else. Some nurses and doctors already understand the language a little and try to explain.”*

A lack of privacy at the time the information is provided to the adolescent was also identified as a problem. One male mystery client narrated the following situation during a focus group session in the rural community:

*“The doctor gave us the information at the hospital entrance, not in a private place. At the end [of the corridor], the doors were open; there were several women who were perhaps waiting for consultation or asking for medication. She then explained to us the methods they had. We felt ashamed because she received us outside; she didn’t take us to a more discreet place. Finally, she explained to us how the intakes [of the contraceptive pill] were going to be.”*

It is common for staff—from security employees to medical personnel—to be members of the same community as the adolescents. For this reason, young people have qualms about the lack of discretion and confidentiality; they also fear being judged. “For women,” affirmed an urban female teacher at an interview, “it’s harder to come because of the shame: ’what are they going to think of me, that I’m no good?‴

Another problem concerned the limited access to contraceptive methods resulting from shortages. In the words of a health professional interviewed at an urban health facility: "Shortages are seen as ‘lost opportunities’… because, sometimes, we already had the session, the girl is convinced and decides, for example, to use an implant but, sometimes, it turns out that there aren’t any."

Finally, with regards to accessibility of care, a female urban/rural community leader indicated at an interview that service hours were incompatible with adolescent schedules: “adolescents are in school when health services are open.”

### Transversal factors across levels

Although we already described the factors that were identified within each level of the socio-ecological framework, there are that converge in more than one level (Table 5). Considering premarital sex unacceptable and lack of familiarity with contraceptive methods are observed at the individual, family, school, and community levels. While other factors are present in three of the four observed levels: ideas that prevent gender equality, access to CSE and to a free and informed exercise of sexuality. The foregoing leads to clandestine dating relationships, risky sexual behaviors, child marriages, school dropouts, and the normalization of violence. Finally, we identified that when two levels converge, there are barriers that impede the development of skills for searching information, access to contraception, and negotiation with the partner. We also observed that autonomous sexual and reproductive exercise is inhibited (Table 5).

**Table 5:** Values, perceptions and practices regarding adolescent sexual and reproductive health according with socio ecological model levels.

Values, perceptions and practices	Levels*			
	I	F	S	C
Having a sexual life before union/marriage is unacceptable	X	X	X	X
Adolescents are not allowed to familiarize themselves with or access modern contraceptive methods	X	X	X	X
Female adolescents often drop out of school when they begin dating or because family chores are prioritized	X	X	X	
Cultural values in favor of early marriage (at the onset of menstruation) have been internalized and normalized	X	X		X
Many are embarrassed to address sexuality-related issues with the health and academic personnel because they sometimes live in the same or a nearby community, and thus fear being exposed	X		X	X
Gender inequality is promoted as regards access to education, health services and the development of social skills that favor sexual and reproductive health	X		X	X
Public displays of affection are prohibited	X		X	X
Clandestine relationships are common	X		X	X
Dating is not accepted as a form of relationship prior to marriage or union between adolescents		X	X	X
Adults feel that providing sexuality information to adolescents hastens the initiation of their sexual lives		X	X	X
Emphasis is placed on risks rather than the exercise of one’s rights		X	X	X
Situations of violence are normalized		X	X	X
Women are more susceptible than men to harassment, abuse, and violence		X	X	X
Adults are reluctant to address CSE, eg, the perspective of rights		X	X	X
Equal opportunities are not offered for both sexes, discriminating mainly against women		X	X	X
Formal education for men is prioritized; women receive less support to stay in school and are made responsible for domestic work		X	X	X
Cultural values (customs and conventions) are based on myths, taboos and prejudices		X	X	X
Female adolescents need to develop the necessary skills to speak, negotiate and agree with their partners on the exercise of their sexuality and reproduction	X	X		
Parents attach little importance to sexuality education and do not encourage seeking care for the sexual and reproductive health of their sons and daughters		X	X	
Teachers and parent committees are reluctant to introduce the topic of sexuality in the classroom		X	X	
Adolescents are not allowed to access sexual and reproductive health services freely		X		X
Parents decide who their children should marry or live in union with; adolescents have no freedom or autonomy in decisions regarding who to associate with and when		X		X
Practices such as forced marriages and/or buying and selling of girls and adolescent women are allowed		X		X
If a female adolescent regret having left home to marry or live in union with a partner, she is practically forbidden to return		X		X
Once adolescents leave home to marry or live with a partner, they are pressured by their parents and in-laws to become pregnant soon and consolidate the marital relationship with the birth of a child		X		X
There are no opportunities to become knowledgeable about contraceptive methods in order to make informed decisions on family planning, eg, when to have children, with whom and how many		X		X

* I: Individual; F: Family; S: School; C: Community

CSE, comprehensive sexuality education

## Discussion

This study explored the contexts and needs for early-pregnancy prevention in two predominantly indigenous communities in Chiapas. An ecological perspective was adopted to analyze the prevention of adolescent pregnancy and the exercise of sexual and reproductive rights. Using a combination of quantitative and qualitative methodologies and a relatively large sample of young adolescents allow us to identify a series of socio-cultural, family, educational and individual factors that impact the lives of adolescents and hinder their ability to recognize themselves as subjects of rights; these same factors also undermine their capacity to exercise the right to make informed decisions regarding pregnancy prevention.

Results demonstrate that adolescents in vulnerable contexts are aware of only a limited number of sexual and reproductive rights. Among these, the least recognized are those related to autonomy and the right to enjoy a satisfying sexual life. Those speaking an indigenous language and living in overcrowded households or in rural areas were found to be the least knowledgeable. Previous research has shown that partial or complete unawareness of sexual and reproductive rights prevents people from enjoying the full benefits of citizenship [29, 30]. In addition, as has been observed in other countries [31], lacking awareness of the right to information and to SRH services carries a whole set of negative consequences for the health of adolescents, often culminating in domestic violence.

As in most studies, our findings too confirm that, except for the male condom, adolescents are generally unaware of contraceptive methods that are safe and effective among this population [17, 18]. However, the information already available does not indicate whether knowledge of contraceptive methods is useful in practice. In Mexico, 98.1% of adolescents have heard of contraceptives; however, of these, only 92.4% know how to use them [32]. In addition, our results indicate that it is essential to provide information on the places where the methods are available: only half of the adolescents who were familiar with male condoms knew where they could be obtained. The situation is even worse in the case of IUDs: only one in six knew where to find them. It is therefore crucial to ensure that readily available information is coupled with easily and timely access to contraceptive services and methods—particularly the more effective ones such as long-acting reversible contraceptives [33]. Such efforts must place more emphasis on marginalized populations whose members have lower levels of access to both information and methods for preventing pregnancy. Our results showed that speakers of indigenous languages and those living in overcrowded conditions were less knowledgeable about contraceptive methods than other participants. Previous studies have shown a similar disadvantage for people living in poverty [17, 18]. Given that adolescents frequently consult social networks and other mass media for information, innovative strategies to render evidence-based platforms widely accessible to all adolescents, even those most marginalized, could be an effective way of helping them acquire accurate knowledge concerning not only contraception [34].

Consistent with other studies, the present study results indicate that the existence of many taboos and prejudices in the family environment inhibits open discussion of sexuality. This reticence is rooted in culturally entrenched and standardized norms and values within the communities studied, rendering it difficult for parents to establish adequate and comfortable avenues of communication with their children concerning sexuality. Several studies have confirmed the central role that parents can play when they talk frequently and frankly with their children about sexuality and health issues. Adolescents who discuss these topics with their parents have generally been found to be more knowledgeable about pregnancy, methods of self-care and the prevention of sexually transmitted infections [35–37]. Developing strategies to enhance awareness and facilitate training in communication concerning sexuality is crucial to ensuring that parents have accurate and timely information that they can pass onto their children [38].

Schools are consistently referred ad the main source of information for adolescents on contraceptives. However, this study uncovered significant barriers among teachers that need to be dealt with, in particular, a lack of training for discussing these issues. This problem is not exclusive to the highlands of Chiapas, as other studies have reported the difficulties teachers encounter in raising issues of sexuality [38, 39]. Additionally, talking about sexuality with students can lead to conflicts with parents, a finding also substantiated by research. The evidence is clear that when teachers discuss the correct use of contraceptives and where to obtain them, the incidence of adolescent pregnancy decreases significantly [35]. It is therefore essential that they receive training on how to address these issues and transmit the information in an appropriate manner. Such training must be accompanied by community-level strategies that enhance the awareness of adults, in order to avoid conflicts in the delivery of the information.

This study found that cultural norms in these communities permeate the lives of adolescents, undermining the ability to fully exercise their sexual and reproductive rights. The study findings, in line with other studies conducted in indigenous communities in Mexico, show that norms anchored in a patriarchal vision of gender relations manifest as a lack of respect for personal autonomy, resulting in premature marriages and early pregnancies [13].

Regarding the provision of SRH services for the adolescent population, the lack of intercultural and youth perspectives—as well as a dearth of training for health professionals in communications, counseling and offering quality care—is identified in this study. Mexico has endeavored to improve coverage for SRH services. On August 12, 2015, the Official Mexican standard for the healthcare of the age group 10–19 years (NOM-047-SSA2-2015) was published in the official journal of the federation. This standard establishes that adolescents may request counseling from health personnel on family planning, sexual and reproductive health, contraceptive methods, prevention of unplanned pregnancy and prevention of sexually transmitted infections [40]. It also states that during counseling adolescents are not required to be accompanied by their mother, father, guardian or legal representative. In addition, although it is recognized that adolescent-friendly services must be “accessible, equitable, acceptable, appropriate, comprehensive, effective and efficient” [4], the findings from this study showed that services with these characteristics are not widely available or provided in a consistent manner to the entire adolescent population. These deficiencies are particularly pronounced in the case of those facing increased social disadvantages, such as the members of the communities studied here. Continuing education programs must therefore be implemented for health personnel who work with adolescents, especially those living in vulnerable contexts.

In 2015, the Mexican government implemented the National Strategy for the Prevention of Adolescent Pregnancy an interinstitutional program whose main objective is to reduce the number of adolescent pregnancies through the promotion of comprehensive sexuality education to strengthen the capacities of boys, girls, and adolescents to fully exercise their rights. This strategy calls for a targeted and efficient strategy designed for those who live in precarious conditions, as well as people living in rural, indigenous and Afro-Mexican communities, as these populations present the most distressing indicators. In the specific case of indigenous communities, the period between 2014 and 2018 witnessed an increase in national adolescent fertility rate, which rose from 84.7 to 87.1 births per 1,000 adolescents [6]. Accordingly, efforts towards achieving recognition and respect for adolescent SRH and rights, represent an important aspect of public health, as ensuring these rights are implemented in their lives, exerting a positive impact on women’s health indicators and favoring sustainable development at the national level [39].

This study has some limitations. Our results are not generalizable to the whole country, because we prioritize collecting detailed information in a specific context, to have a deep understanding of the sexual and reproductive health needs in a well-characterized setting. However, the value of qualitative research does not lie in the statistical representation of its findings but in the possibility of deepening the understanding of the social processes studied. Since our interest was to investigate and present an exploratory and descriptive analysis of perceptions and experiences in relation to adolescent pregnancy in the context of Chiapas, we did not conduct a comparative analysis by actor and type of community. We focused on having a diverse sample. Our study is also prone to selection bias, given the self-selection of volunteers to participate in the study. We implemented self-administered questionnaires using a computerized audio system (ACASI) to reduce the risk of errors during application, and to ensure privacy and confidentiality.

## Conclusions

The exercise of sexual and reproductive rights in adolescents in vulnerable contexts face several challenges at different levels of analysis such as lack of knowledge and tools in sexual and reproductive health among healthcare personnel, teachers, parents, and adolescents themselves, limited space for adolescent counseling services, framed in beliefs and customs that restrict autonomy in the exercise of adolescent sexuality. Therefore, it is necessary to guarantee reproductive justice in rural and indigenous contexts where social, cultural, and economic barriers to exercise sexual and reproductive rights are recognized and eliminated. Additionally, it is essential to implement policies focused on adolescence as a critical stage of development between childhood and adulthood, where the perspective of human rights, intercultural and health-equity are incorporated. Such an approach must consider multi-components to be effective to enhance knowledge, foster adolescent empowerment and ensure access to improved SRH care, including the benefits of postponing pregnancy.

## Supporting information

S1 Checklist PRISMA statement—checklist of items that should be included in reports of observational studies. (DOCX)

S1 Text Quantitative Spanish questionnaires. (PDF)

S1 Data Stata file of the summarized data extracted. (DTA)
