# Supplementary material for: Sexual and reproductive health among adolescents in vulnerable contexts in Mexico: Needs, knowledge, and rights
Source: PLOS Glob Public Health. 2023 Nov 1;3(11):e0002396. doi: 10.1371/journal.pgph.0002396 (PMC10619806; doi:10.1371/journal.pgph.0002396)
Supplement: S1 Checklist — (DOCX) [file pgph.0002396.s001.docx]

PRISMA Statement—checklist of items that should be included in reports of observational studies

|  | Item No. | Recommendation | Page  No. | Relevant text from manuscript |
| --- | --- | --- | --- | --- |
| **Title and abstract** | 1 | (*a*) Indicate the study’s design with a commonly used term in the title or the abstract | 2 | Using a convergent parallel mixed-methods study design |
|  |  | (*b*) Provide in the abstract an informative and balanced summary of what was done and what was found | 2 | Results showed that adolescents recognized their right to receive sexuality education |
| Introduction | | | |  |
| Background/rationale | 2 | Explain the scientific background and rationale for the investigation being reported | 3 & 4 | Indigenous women experience dramatic SRH inequities [13–15] as well as barriers to information and restricted access to contraceptive methods |
| Objectives | 3 | State specific objectives, including any prespecified hypotheses | 4 | We aimed to identify the healthcare requirements that would support efforts to prevent adolescent pregnancy in two predominantly indigenous communities in Chiapas  Hypotheses NA |
| Methods | | | |  |
| Study design | 4 | Present key elements of study design early in the paper | 4 | [The ecological] model allowed us to visualize of the complex factors underlying adolescent pregnancy **(Figure 1)**. |
| Setting | 5 | Describe the setting, locations, and relevant dates, including periods of recruitment, exposure, follow-up, and data collection | 5 | We undertook the study in Chiapas |
| Participants | 6 | (*a*) *Cohort study*—Give the eligibility criteria, and the sources and methods of selection of participants. Describe methods of follow-up  *Case-control study*—Give the eligibility criteria, and the sources and methods of case ascertainment and control selection. Give the rationale for the choice of cases and controls  *Cross-sectional study*—Give the eligibility criteria, and the sources and methods of selection of participants | 5 & 6 | Adolescents (12-15 years old) in the second and third years of lower secondary school completed a self-administered questionnaire. Participants included medical, nursing, psychology, and social-work staff as well as community-level promoters involved in adolescent SRH activities. Those providing primary- and secondary-healthcare services to adolescents completed an online, self-administered survey |
|  |  | (*b*) *Cohort study*—For matched studies, give matching criteria and number of exposed and unexposed  *Case-control study*—For matched studies, give matching criteria and the number of controls per case |  |  |
| Variables | 7 | Clearly define all outcomes, exposures, predictors, potential confounders, and effect modifiers. Give diagnostic criteria, if applicable | 7 | we constructed two dichotomous variables in order to identify the most knowledgeable adolescents, that is, those with the greatest amount of information on their sexual and reproductive rights (above average: >3 rights) and on contraceptive methods (above average: >2 contraceptive methods). |
| Data sources/ measurement | 8* | For each variable of interest, give sources of data and details of methods of assessment (measurement). Describe comparability of assessment methods if there is more than one group | 31 | Figure 2 |
| Bias | 9 | Describe any efforts to address potential sources of bias | 6 & 7 | In order to reduce social desirability bias and ensure privacy, we used an audio-computer assisted self- interview (ACASI) method […]  We ensured data variability by incorporating the perspectives of diverse social actors, collecting information from different sites, and integrating a variety of topics in the objective |
| Study size | 10 | Explain how the study size was arrived at | 5 | Since this is an observational, exploratory study, we did not perform sample size calculations. Once we selected the communities, we choose the schools with the largest number of students, assuming that this would allow us to have a greater variability in our population. |

Continued on next page

| Quantitative variables | 11 | Explain how quantitative variables were handled in the analyses. If applicable, describe which groupings were chosen and why | 7 | we constructed two dichotomous variables in order to identify the most knowledgeable adolescents, that is, those with the greatest amount of information on their sexual and reproductive rights (above average: >3 rights) and on contraceptive methods (above average: >2 contraceptive methods). |
| --- | --- | --- | --- | --- |
| Statistical methods | 12 | (*a*) Describe all statistical methods, including those used to control for confounding | 7 | We adjusted a multivariate logistic-regression model to explore the association of each sociodemographic variable with knowledge regarding sexual and reproductive rights and contraceptive methods |
|  |  | (*b*) Describe any methods used to examine subgroups and interactions |  | NA |
|  |  | (*c*) Explain how missing data were addressed |  | NA |
|  |  | (*d*) *Cohort study*—If applicable, explain how loss to follow-up was addressed  *Case-control study*—If applicable, explain how matching of cases and controls was addressed  *Cross-sectional study*—If applicable, describe analytical methods taking account of sampling strategy |  | NA |
|  |  | (*e*) Describe any sensitivity analyses |  | NA |
| Results | | | | |
| Participants | 13* | (a) Report numbers of individuals at each stage of study—eg numbers potentially eligible, examined for eligibility, confirmed eligible, included in the study, completing follow-up, and analysed | 7 & 31 | Figure 2 presents the characteristics of the participating adolescents, mothers and fathers of adolescents, schoolteachers, health personnel, young women with a history of adolescent pregnancy, and community leaders who participated in the study |
|  |  | (b) Give reasons for non-participation at each stage |  | NA |
|  |  | (c) Consider use of a flow diagram |  | NA |
| Descriptive data | 14* | (a) Give characteristics of study participants (eg demographic, clinical, social) and information on exposures and potential confounders | 7,8 & 32 | A total of 911 adolescents of mean (standard deviation [SD]) age 13.8 (0.8) years were surveyed; of these, 50.8% were male, 24.0% spoke an indigenous language, 5.4% resided in rural areas and 12.6% lived in overcrowded households (more than 2.5 people per room). In total, 12 health personnel of mean (SD) age 44 (6.7) were surveyed; of which, 91.7% were female and 58.3% worked in health units **(Table 1)**. |
|  |  | (b) Indicate number of participants with missing data for each variable of interest |  | NA |
|  |  | (c) *Cohort study*—Summarise follow-up time (eg, average and total amount) |  | NA |
| Outcome data | 15* | *Cohort study*—Report numbers of outcome events or summary measures over time |  | NA |
|  |  | *Case-control study—*Report numbers in each exposure category, or summary measures of exposure |  | NA |
|  |  | *Cross-sectional study—*Report numbers of outcome events or summary measures | 8, 33 & 34 | Table 2  Regarding knowledge on contraception, adolescents were acquainted with an average of three contraceptive methods, with most respondents having heard of male condoms (95.6%) and contraceptive pills (86.4%). |
| Main results | 16 | (*a*) Give unadjusted estimates and, if applicable, confounder-adjusted estimates and their precision (eg, 95% confidence interval). Make clear which confounders were adjusted for and why they were included | 36 | Table 3  The cross-sectional design of the study does not allow for assessing causal inferences between a particular characteristic and our outcomes (increased knowledge of contraceptive methods and increased knowledge of sexual and reproductive rights). |
|  |  | (*b*) Report category boundaries when continuous variables were categorized | 36 | Table 3 |
|  |  | (*c*) If relevant, consider translating estimates of relative risk into absolute risk for a meaningful time period |  | NA |

Continued on next page

| Other analyses | 17 | Report other analyses done—eg analyses of subgroups and interactions, and sensitivity analyses |  | NA |
| --- | --- | --- | --- | --- |
| Discussion | | | | |
| Key results | 18 | Summarise key results with reference to study objectives | 17 | Using a combination of quantitative and qualitative methodologies and a relatively large sample of young adolescents allow us to identify a series of socio-cultural, family, educational and individual factors that impact the lives of adolescents and hinder their ability to recognize themselves as subjects of rights; these same factors also undermine their capacity to exercise the right to make informed decisions regarding pregnancy prevention. |
| Limitations | 19 | Discuss limitations of the study, taking into account sources of potential bias or imprecision. Discuss both direction and magnitude of any potential bias | 21 | This study has some limitations. Our results are not […] |
| Interpretation | 20 | Give a cautious overall interpretation of results considering objectives, limitations, multiplicity of analyses, results from similar studies, and other relevant evidence | 17 to 21 | As in most studies, our findings too confirm that, except for the male condom, adolescents are generally unaware of contraceptive methods that are safe and effective among this population [16,17].  This problem is not exclusive to the highlands of Chiapas, as other studies have reported the difficulties teachers encounter in raising issues of sexuality [36,37]. |
| Generalisability | 21 | Discuss the generalisability (external validity) of the study results | 21 | […]. Our results are not generalizable to the whole country, […] |
| Other information | |  | | |
| Funding | 22 | Give the source of funding and the role of the funders for the present study and, if applicable, for the original study on which the present article is based | 22 | This research was funded by Organon & Co. The funder had no role in the development, conduct, or interpretation of the present study. |

*Give information separately for cases and controls in case-control studies and, if applicable, for exposed and unexposed groups in cohort and cross-sectional studies.

**Note:** An Explanation and Elaboration article discusses each checklist item and gives methodological background and published examples of transparent reporting. The STROBE checklist is best used in conjunction with this article (freely available on the Web sites of PLoS Medicine at http://www.plosmedicine.org/, Annals of Internal Medicine at http://www.annals.org/, and Epidemiology at http://www.epidem.com/). Information on the STROBE Initiative is available at www.strobe-statement.org.
