# Supplementary material for: Sexual and reproductive health among adolescents in vulnerable contexts in Mexico: Needs, knowledge, and rights
Source: PLOS Glob Public Health. 2023 Nov 1;3(11):e0002396. doi: 10.1371/journal.pgph.0002396 (PMC10619806; doi:10.1371/journal.pgph.0002396)
Supplement: S1 Text — (PDF) [file pgph.0002396.s002.pdf]

Instituto Nacional de Salud Pública

CUESTIONARIO AUTOAPLICABLE
FAVOR DE NO CONTESTAR ESTA HOJA

Diagnóstico sobre necesidades en salud sexual y reproductiva para la prevención del embarazo en la adolescencia en comunidades de Chiapas

I. IDENTIFICACIÓN GEOGRÁFICA

ENTIDAD FEDERATIVA ______________________ |___|___|

MUNICIPIO O DELEGACIÓN ______________________ |___|___|___|

LOCALIDAD ______________________ |___|___|___|___|

ESTRATO ______________________ |___|___|___|

AGEB ______________________ |___|___|___|___|

II. FOLIO

|___|___|___|___|___|___|___|___|___|

III. DIRECCIÓN DE LA ESCUELA SECUNDARIA

DOMICILIO: ______________________

COLONIA: ______________________

C.P.: ______________________

TELÉFONO DE LA ESCUELA: ______________________

NOMBRE DEL (A) DIRECTOR (A): ______________________

IV. DATOS DEL ALUMNO

GRADO QUE CURSA______________|___|___|

CLAVE DEL GRUPO______________|___|___|

MATUTINO 1

1. DATOS GENERALES

Las siguientes son preguntas sobre tu familia, y de la vivienda donde habitas, por favor marca la respuesta correcta.

1.1 ¿Cuántos años cumplidos tienes?	Años cumplidos………………I___I___I	
1.2 ¿Eres	Hombre? ...…………………………………………1<br>Mujer? ……………………………………………...2	
1.3 ¿Con quién vives?<br><br>**<u>Puedes marcar más de una opción</u>**	Tus padres…………………………………………1<br>Sólo mamá / sólo papá………………………......2<br>Hermanos (as)……………………...……...…3<br>Otros familiares………………………………...…4<br>Otra personas que no son familiares………..…5<br>Solo (a)………………………………………..……6<br>Con amigos………………………………………...7<br>Otro ________________________________8<br>(especifique)	
1.4 ¿Cuántas personas viven en tu casa?	I_____I_____I	
1.5 En tu casa, ¿cuántos cuartos se usan para dormir sin contar pasillos ni cocina?	I_____I_____I Cuartos de dormitorio	
1.6 ¿Hablas alguna lengua indígena?	Sí……………………………………………………1<br>No…………………………………………………..2	**2 ⟶ Pasa a 1.8**
1.7 En casa, tú y tu familia la mayor parte del tiempo hablan:	Español……………………………………………1<br>Lengua indígena…………………………………2	
1.8 ¿Cuál es tu religión?<br><br>**<u>Sólo marca una opción de respuesta</u>**	Ninguna ………………………….......……………1<br>Católica.......…...........………………………....2<br>Testigo de Jehová ....3<br>Adventista del 7° día ……..………………………4<br>Evangelista ....…5<br>Pentecostés…………..……….………………..6<br>Mormón ....7<br>Otra………………………………….……………..8<br>No quiero contestar..9	**1 ⟶ Pasa a 1.10**<br><br>**9 ⟶ Pasa a 1.10**
1.9 ¿Con qué frecuencia asistes a la Iglesia/Templo?	Diario………………………………………………..1<br>Por lo menos una vez por semana………………2<br>Por lo menos una vez al mes …………………….3<br>Por lo menos una vez al año………………………4<br>Menos de una vez al año …………………………5<br>Nunca……………………………………………….6	
1.10 ¿A qué te dedicas?	Sólo estudio……………………...………….……1<br>Estudio y trabajo ……………………………..…….2	

1.11 Cuando te enfermas, ¿a dónde acudes por ayuda?	Doctor en centro de salud, clínica, hospital (ISSSTE, SSA, IMSS Oportunidades), clínica pública……………………………………………1<br>Consultorio/clínica privada.…….……….........2<br>Consultorio en una Farmacia………………..…3<br>Curandero/hierbero………. …………….…….4<br>No voy a ninguna parte……………………...…5<br>No sé……………………………………………6	
1.12 ¿Alguna vez has utilizado internet?	Sí…………….…………………….……………1<br>No…………………………………….………..2	**2 → Pasa a 2.1**
1.13 Durante los últimos 3 meses, ¿qué tan seguido usas internet?	Nunca……………….……………….………....0<br>Menos de una vez por semana………………...1<br>Al menos una vez por semana……..…………..2<br>Casi todos los días….………… ……………..…3	
1.14. ¿Tienes teléfono celular o tableta con internet?	Sí, sólo lo uso yo……………………………….…1<br>Sí, lo comparto con mi familia…………………..2<br>No tengo.………………………………………...3	

1.15 Indica si has usado alguna de las siguientes opciones			
	1) Si lo he usado	2) No lo he usado	No lo conozco
Facebook			
WhatsApp			
Youtube			
Juegos en internet			
Otro_________________________________			

2. CONOCIMIENTOS SOBRE REPRODUCCIÓN Y MÉTODOS ANTICONCEPTIVOS

A continuación, nos referiremos a los métodos o medios que puede usar una pareja para evitar o planear un embarazo.

2.1¿Has oído hablar de (Método)…<br><br>Sí ………………………..………………..1<br><br>No……………………………….……..….2 → Pasa al siguiente inciso	2.2 ¿Conoces algún lugar o persona donde las personas/gente de tu edad puedan obtener (Método del inciso de 2.1)?<br><br>Sí…………………..……….1<br><br>No………………………2	2.3 ¿Dónde escuchaste de la existencia de (Método del inciso 2.1)?<br>Escuela .... 1<br>Casa .... 2<br>Con el Dr. o Clínica u Hospital ..... 3<br>Internet .... 4<br>**Novio o pareja**…………………….. 5<br>Amigos .... 6<br>Otro lugar…………………………..7
a) las pastillas anticonceptivas? I___I	I___I	I___I
b) **DIU** I___I	I___I	I___I
c) implante I___I	I___I	I___I
d) del condón? I___I	I___I	I___I
e) la pastilla de anticoncepción de emergencia? (del día después) I___I	I___I	I___I

2.4 ¿En qué momento del ciclo menstrual es más probable que una mujer quede embarazada?	Un día antes de que la regla comience …………1<br>Cinco días después de que la regla comenzó …2<br>Catorce días después de que la regla comenzó..3<br>No hay diferencia, cualquier momento es igual…4	

	No sé……………………………………………………5	
2.5 ¿Cuántas veces se puede usar un condón masculino?	I___I veces<br>No sé...88	
2.6 ¿Alguna vez has tenido un condón en tus manos?	Si ……………………………………………………1<br>No……………………………………………………2	
2.7 El condón se utiliza para…	Prevenir un embarazo..1<br>Prevenir una infección de transmisión sexual .....2<br>Las dos cosas..3<br>No lo sé...8<br>No quiero contestar ……………………….…99	
2.8 ¿En qué momento se debe poner el condón masculino?	Cuando se ha eyaculado…………………………1<br>Cuando el pene está erecto………………………2<br>Antes de que el pene esté erecto………………..3<br>No sé………………………………………………..4	
2.9 Los condones pueden zafarse y quedarse adentro de la mujer	Sí……………………………………………………1<br>No…………………………………………………..2	
2.10 ¿Una mujer puede quedar embarazada la primera vez que tiene relaciones sexuales?	Sí……………………………………………………1<br>No…………………………………………………..2<br>No sé ………………………………………………3	
2.11 ¿En dónde escuchaste hablar de algún método para no embarazarse o no tener hijos?	Escuela……………………………………………1<br>Familia……………………………………………2<br>Centro de salud o médico………………………3<br>**Novio o pareja**………………………………… 4<br>Farmacia…………………………………………5<br>Internet…………………………………………...6<br>Amigos……………………………………………7<br>Otro………………………………………………8<br>Nadie me ha hablado de métodos anticonceptivos…………………………………9	
2.12 ¿De quién has recibido información sobre temas de sexualidad y prevención de embarazo?	Maestro/a…………………………………………1<br>Madre o padre……………………………………2<br>Hermanos/hermanas……………………………3<br>**Novio o pareja**………………………………… 4<br>Amigos ……………………………………………5<br>Internet……………………………………………6<br>Libros/revistas……………………………………7<br>Talleres, curso……………………………………8<br>Películas/TV………………………………………9	
2.13 ¿Alguna vez has consultado una página de internet para informarte sobre cuestiones de sexualidad y/o prevención o salud sexual?	Sí……………………………………………………1<br>No…………………………………………………..2	

2.14 ¿Cuáles de los siguientes métodos crees que sirvan para prevenir una infección de transmisión sexual o VIH y/o SIDA<br><br>**<u>Puedes marcar más de una opción</u>**	Retiro o venirse afuera? …….……………………..1<br>Pastillas o píldoras? ……..……..……..……….....2<br>Lavado vaginal? ………………………………......3<br>Condones? ……………………………..………4<br>Ritmo o calendario? …………………………..…..5<br>Tomar té de hierbas? ……………………..……6<br>DIU…………………………………………………..7<br>Relaciones sólo con tu pareja? ……..……………8<br>No tener relaciones sexuales?………..……......9<br>Otro?..……......77	

3. AUTO-EFICACIA

A continuación, se te presentan algunas situaciones, que preguntan sobre si crees que podrías hacer las siguientes cosas. No hay respuestas correctas ni incorrectas.

3.1¿Crees que podrías…		Sí	Tal vez	No
a)	Hablar sobre usar condón con la persona con la que quisieras tener relaciones sexuales?			
b)	Identificar ventajas y desventajas para decidir cuándo y con quién tener relaciones sexuales?			
c)	Negarte a tener sexo cuando no quieres?			
d)	Hablar con tu novio/a **o pareja** sobre la forma de evitar un embarazo e infecciones de transmisión sexual?			
e)	Convencer a **tu novio/a o** pareja de usar un condón cuando tengan relaciones sexuales?			
f)	Decirle a tu **novio/a o** pareja que no vas a tener relaciones sexuales si no quiere usar condón?			
g)	Usar correctamente el condón en todas tus relaciones sexuales?			
h)	Ir a la farmacia o a la tienda a comprar condones?			
i)	Ir a la clínica o al centro de salud a pedir condones e información sobre métodos anticonceptivos?			
j)	Usar condón junto con otro método anticonceptivo (como DIU, parches o implantes), para tener doble protección?			
k)	Usar un condón nuevo cada vez que tengas relaciones sexuales con tu pareja?			
l)	Ponerte un condón a ti mismo o a tu pareja antes de la penetración?			

4. USO DE SERVICIOS DE SALUD SEXUAL Y REPRODUCTIVA

Las siguientes preguntas están relacionadas con el uso de servicios de salud sexual y reproductiva y el acceso a condones.

4.1 ¿Alguna vez has ido a un centro de salud o clínica para pedir información o servicios sobre anticonceptivos, embarazo o enfermedades transmitidas sexualmente?	Sí……………………………………………..……1<br>No……………………………………..…………2	**2 ⟶Pasa a 4.6**
4.2 En el centro de salud, ¿te habló sobre…<br><br>**<u>Puedes seleccionar más de una opción</u>**	a) anticonceptivos?..1<br>b) condones?...2<br>c) anticoncepción de emergencia o pastilla del día después? ....3<br>d) enfermedades transmitidas sexualmente? ….4<br>e) el embarazo? 5	
4.3 ¿Pediste anticonceptivos?	Sí……………………………………………..……1<br>No……………………………………..…………2	
4.4 ¿Te dio pena/ te sentiste incómodo(a) para hacer preguntas?	Sí……………………………………………..……1	

	No……………………………………………………2	2 → Pasa a 4.7
4.5 ¿Te contestaron tus preguntas y te resolvieron tus dudas de manera adecuada durante la consulta?	Sí……………………………………………………1<br>No……………………………………………………2	
4.6 Alguna vez has pensado en ir a un servicio de salud para…<br><br>**<u>Puedes elegir más de una opción</u>**	Pedir información sobre métodos anticonceptivos…………………………………1<br>Pedir un método anticonceptivo…………………2<br>Revisar un método anticonceptivo que uso ……..3<br>Nunca he pensado en ir a un centro de salud.….4	4 → Pasa a 4.10
4.7 En los últimos 12 meses, en el centro de salud ¿has recibido condones de forma gratuita?	Sí……………………………………………………1<br>No……………………………………………………2<br>No sé…………………………………………………..8	2<br>8 } Pasa a 4.10
4.8 Cuando fuiste al centro de salud, ¿alguna vez te negaron o te dijeron que no te podían dar el servicio de planificación familiar?	Sí……………………………………………………1<br>No……………………………………………………2	2 → Pasa a 5.1
4.9 ¿Qué te dijeron cuando te negaron el servicio?	Todavía no tenía edad para usar anticonceptivos….1<br>Que llevara a algún adulto ....2<br>No tenían anticonceptivos ....3<br>No había personal de salud ....4<br>Estaba cerrada la institución ....5<br>Otro (especifique)<br>_____________________..................................... .6	Pasa a 5.1
4.10 La razón principal por qué no has ido a solicitar anticonceptivos o asesoría al centro de salud es…	Pena……………………………………………………1<br>No se me ha ocurrido……………………………...2<br>No lo necesito……………………………………3<br>Todos me conocen y le dirían a mi mamá…………4<br>No te hacen caso……………………………………5<br>Otra (especifique)<br>___________________________........................6	

5. INICIO DE VIDA SEXUAL

Las siguientes preguntas están relacionadas con tu experiencia sexual, recuerda que tus respuestas son estrictamente confidenciales.

5.1 ¿Has tenido alguna vez relaciones sexuales?	Sí ………………………………………………..1<br>No……………………………………………...2	2 → Pasa a 5.15
5.2 ¿A qué edad tuviste tu primera relación sexual?	Edad……………………………I___I___I<br>No me acuerdo…………………………………...88<br>No quiero contestar ………………………………...99	
5.3 ¿Con quién tuviste tu primera relación sexual?	Con mi novio(a)………………………………….1<br>Con un amigo(a)……………………………….....2<br>Con un familiar……………………………………..3<br>Con un trabajador(a) sexual/prostituto(a).………4<br>Con un maestro(a)……………………………..5	

	Con mi esposo(a)……………………….….……...6<br>Con un desconocido(a)……………….……......7<br>Otro ________________________________ 8<br>(especifique)<br>No quiero contestar ……………………………99	
5.4 ¿Qué edad tenía la persona con la que tuviste tu primera relación sexual?	Edad……………………………I___I___I<br>No me acuerdo ……………………………..…...87<br>No sé…………………. ………………..……..88	
5.5 ¿Cuándo tuviste esa primera relación sexual, habías tomado alcohol?	Sí…………………………………………………....1<br>No………………………………………………...2	
5.6 En tu primera relación sexual, ¿usaron algún método anticonceptivo?	Sí…………………………………………………....1<br>No………………………………………………...2	2 → Pasa a 5.8
5.7 ¿Qué método anticonceptivo usaron?	Condón ……………………………………..……1<br>Pastillas de anticoncepción de emergencia o del día siguiente……………………………………3<br>Pastillas anticonceptivas………..……………4<br>Dispositivo, DIU o aparato………………………6<br>Implantes, tubos o norplant……………………..7<br>Parche anticonceptivo…………………………..8<br>Otro (especifique) _______________...........14<br>No recuerda……………………………………..77<br>No sé……………………………………………88	Pasa a 5.9
5.8 ¿Por qué no utilizaron **algún** método?	No sabíamos dónde conseguirlo…………..…....1<br>No lo planeamos…………………..……………...2<br>No teníamos dinero……………………..………3<br>No sabíamos que existían………….…..……..4<br>No sabíamos usarlo…………………...……….5<br>Teníamos pena para usarlo………………..……6<br>La otra persona(a) no quiso usarlo……….……7<br>Yo no quise usarlo…………………….…..……...8<br>No pensé que nos fuéramos a embarazar………10<br>Porque fue una relación sexual con alguien del mismo sexo………………..…..……....………...11<br>Otro ……………………………………………..…88	
5.9 ¿Has tenido más de una relación sexual en tu vida?	Sí …………………….…………..……....……….1<br>No…………………………………………...……...2	2 → Pasa a 6.2
5.10 En tu última relación sexual, ¿usaron algún método anticonceptivo?	Sí …………………….…………..……...………..1<br>No…………………………………………...……...2	2 → Pasa a 5.12
5.11 En la última relación sexual, ¿qué utilizaron tú o tu pareja para evitar un embarazo o una infección de transmisión sexual?	Condón ……………………………………..……1<br>Pastillas de anticoncepción de emergencia o del día siguiente……………………………………2<br>Pastillas anticonceptivas………..……………3<br>Dispositivo, DIU o aparato………………………4<br>Implantes, tubos o norplant……………………..5<br>Parche anticonceptivo…………………………..6<br>Otro (especifique) _____________________.7<br>No recuerda……………………………………8	1 → Pasa a 5.14<br>Pasa a 5.13

	No sé……………………………………………88	
5.12 ¿Por qué no utilizaron **algún** método?	No sabíamos dónde conseguirlo………………1<br>No lo planeamos……………………………...2<br>No teníamos dinero……………………………3<br>No sabíamos que existían………………………4<br>No sabíamos usarlo…………………………5<br>Teníamos pena para usarlo………………………6<br>La otra persona no quiso usarlo………………7<br>Yo no quise usarlo…………………………..8<br>No pensé que nos fuéramos a embarazar………9<br>Porque fue una relación sexual con alguien del mismo sexo…………………………..10<br>Otro (especifique)______________________88	**Pasa a 5.14**
5.13 ¿Usaron también condón?	Sí……………………………………………1<br>No……………………………………………2	
5.14 En los últimos tres meses, ¿has tenido relaciones sexuales?	Sí……………………………………………1<br>No……………………………………………..2	**Pasa a 6.1**
**Quienes contestaron "Si haber tenido relaciones sexuales" (opción 1) en la pregunta 5.1, pasan a la pregunta 6.1. Los que contestaron que "No han tenido relaciones sexuales" en la pregunta 5.1, pasan a la 5.15**		
5.15 ¿Piensas que tendrás relaciones sexuales antes de cumplir 20 años?	Sí……………………………………………1<br>No / no lo he pensado……………………………..2	
5.16 ¿Piensas usar condón la primera vez que tengas relaciones sexuales?	Sí……………………………………………1<br>No / no sé……………………………………...2	**Pasa a 6.2**

6. ANTECEDENTE DE EMBARAZO

6.1 ¿Alguna vez has estado embarazada?<br><br>**Hombre**:<br>¿Alguna vez has embarazado a alguien?	Sí……………………………………………1<br>Sí, embarazada actualmente……………………..2<br>No……………………………………………3<br>No sé……………………………………………88<br>No quiero contestar ……………………………..99	**1 → Pasa a 6.5**<br>**2 → Pasa a 6.5**<br>**3**<br>**88** **Pasa a 6.2**<br>**99**
**Mujeres con inicio de vida sexual (pregunta 5.1 código 1) y que no ha estado embarazadas (pregunta 6.1 código 3) continúen, sino pasan a 6.7**		
6.2 ¿Te gustaría tener hijos?	Sí……………………………………………...1<br>No……………………………………………..2<br>No sé……………………………………………..8	**1**<br>**2**<br>**8** **Pasa a 7.1**
6.3 ¿A qué edad piensas que está bien que los **hombres** comiencen a tener relaciones sexuales?	|___|___| años<br>No sé……………………………………………..88	
6.4 ¿A qué edad piensas que está bien que las **mujeres** comiencen a tener relaciones sexuales?	|___|___| años<br>No sé……………………………………………..88	**Pasa a 7.1**
**Continúan a la pregunta 6.5 los hombres y mujeres con inicio de vida sexual y que respondieron a la pregunta 6.1: "Sí" (opción 1 y 2).**		

6.5 ¿Cuántas veces has estado embarazada?<br>*(Considere embarazo actual)*<br>**Hombre**: ¿Cuántas veces has embarazado a alguien?	Número de embarazos |____|____| No quiero contestar ……………….……………99	**Si el número de embarazos = 1, pasa a 6.7b**
6.6 ¿Cuántos hijos nacidos vivos has tenido?	Número de hijos nacidos vivos |____|____|	
6.7 La última vez que te embarazaste…<br>6.7b En el momento en que te embarazaste …<br>**Hombre**: En el momento en que se embarazaron la última vez…	Tú querías embarazarte.......................................1<br>Tú querías esperar más tiempo............................2<br>No querías embarazarte…..................................3<br>No quiero contestar ………………………………..99	**Si viene de 6.7b pase a 6.9, sino continúe a 6.8**
**Filtro: Si 6.8 es mayor que cero seguir, sino pasar a 7.1**		
6.8 Además de las hijas o hijos que has tenido, ¿te gustaría tener otra u otro?	Sí……………………………………….…………….…...1<br>No…………………………………………….………...…2<br>No sé……………………………….……………….8<br>No quiero contestar ……………………………99	**Pasa a 7.1**
**Filtro: Si 6.5=1**		
6.9. Además de la hija o del hijo que esperas ¿te gustaría tener otra u otro?	Sí……………………………………….…………….…...1<br>No…………………………………………….………...…2<br>No sé……………………………….……………….8<br>No quiero contestar ………………………………99	

7. DERECHOS SEXUALES Y REPRODUCTIVOS

7.1 ¿De los siguientes enunciados, selecciona cuáles crees que son Derechos Sexuales y Reproductivos de los adolescentes?<br><br>**<u>Puedes seleccionar varias respuestas</u>**	Recibir educación sexual………………………………1<br>Recibir información sobre métodos anticonceptivos……………………………….……………..2<br>Recibir métodos anticonceptivos en farmacias y/o centros de salud sin ir acompañado de un adulto…..3<br>Tener relaciones sexuales sólo cuando lo deseo…..4<br>Tener una vida sexual placentera……………….……5<br>Decidir cuándo tener hijos…………………………....6<br>Decidir cuándo vivir en pareja……………………......7<br>Decidir con quién tener hijos………………………...8<br>Decidir de forma libre sobre mi cuerpo y mi sexualidad……………………………………….……..9<br>Respeto a mi intimidad y mi vida privada…………10<br>Vivir libre de violencia sexual…………………….....11<br>Ninguna……………………….……………………12<br>No sé…………………………………………………13	

8. INTENCIONES DE VIDA

8.1 ¿Cuáles de los siguientes eventos crees que tú puedas planear?	**Sí** lo puedo planear	No lo puedo planear
a) Cuando terminar mis estudios		
b) Cuando empezar a trabajar		
c) Cuando casarme		
d) Cuando tener hijos		
e) Cuantos hijos tener		

8.2 ¿A qué edad te gustaría?	Antes de los 20 años	Después de los 20 años	No me gustaría	**No sé**
a) Casarme / vivir en pareja				
b) Tener mi primer hijo				
c) Tener un empleo estable				

8.3 ¿Hasta cuándo crees que seguirás estudiando?	Hasta terminar la secundaria………………… .1<br>Hasta terminar la preparatoria……………… .2<br>Hasta terminar la universidad………………. .3<br>Más allá de la universidad (posgrado, maestría, doctorado) ……………………………………. .4	
8.4 En general, ir a la escuela:<br><br>**ELIJA SOLO UNA OPCIÓN**	Me parece interesante…………………………1<br>Me divierte………………………………………2<br>No me gusta……………………………………3<br>Me aburre………………………………………4<br>Me da igual……………………………………..5<br>Otra………………………………………………6	
8.5 ¿Qué tipo de actividad u ocupación te gustaría o imaginas estar haciendo dentro de 10 años?	Seguir estudiando…………………………………1<br>Estar estudiando y trabajando…………………..2<br>Trabajando ejerciendo una profesión…………..3<br>Trabajando sin haber estudiado una profesión..4<br>Dedicándome exclusivamente al hogar………..5<br>Nunca he pensado en eso……………………….6	

9. COMUNICACIÓN DE PADRES E HIJOS

9.1 Con qué frecuencia has hablado con tus papás sobre los siguientes temas…	Nunca	Una sola vez	Pocas veces	Algunas veces	Muchas veces
a. Sexo	0	1	2	3	4
b. Cómo usar condones	0	1	2	3	4
c. Cómo evitar un embarazo	0	1	2	3	4

10. ADICCIONES

En esta sección encontrarás preguntas relacionadas con el consumo de alcohol, tabaco y drogas. Por favor selecciona tu respuesta y recuerda que tus respuestas son estrictamente confidenciales.

10.1 ¿Has fumado por lo menos cien cigarrillos (5 cajetillas) de tabaco durante toda tu vida?	Sí……………………………………………………1<br>No…………………………………………………..2<br>Nunca he fumado………………………………...3<br>No quiero contestar ……………………………99	**3 → Pasa a 10.3**<br>**99 → Pasa a 10.3**

10.2 En los últimos 30 días, ¿has fumado tabaco?	Sí……………………………………….……………..……1<br>No………………………………….…….…………..……2	

La próxima pregunta es sobre si tomas alcohol. Esto incluye tomar cerveza, pulque, vino, brandy, whisky, ron, tequila, coolers, presidencola. No incluye tomar unos sorbitos de vino para fiestas religiosas.

10.3 Durante los últimos 30 días, ¿cuántos días tomaste 5 o más bebidas de alcohol en una ocasión?	**......................** /______/______/ días	**Rango de 0 a 30**
10.4 ¿Alguna vez has probado alguna droga, aunque sea ocasionalmente o sólo en fiestas?	Sí…………………………….……………………….1<br>No…………………………….……………………..…..2<br>No quiero contestar ………….………………... 99	

11. BIENES DEL HOGAR Y CONTACTO

Por último, queremos saber algo sobre los bienes que son de tu propiedad o de alguno de los miembros de tu hogar y tu disposición para seguir ayudándonos.

11.1 ¿Tú o alguien que viva en tu casa tienen…			
	Bienes del hogar	Si	No
a)	automóvil?	1	2
b)	televisión?	1	2
c)	servicio de TV de paga/cable? (Megacable, Izzy, Sky, etc.)	1	2
d)	computadora de escritorio o laptop?	1	2
e)	Internet?	1	2
f)	horno de microondas?	1	2
g)	teléfono de casa?	1	2
h)	teléfono celular?	1	2

Para nosotros tu punto de vista es muy importante:

11.2 En caso de que este estudio lo requiera, ¿te gustaría seguir participando, respondiendo a una entrevista personal y anónima en tu misma escuela, para seguir hablando sobre estos temas?	Sí……………………………………………1<br>No…………………………………………..2<br>No sé………………….…………………3	

Control de captura (   )

FOLIO |_____|_____|_____|_____|_____|_____|_____|_____|_____|

Cuestionario para personal de salud

(Centros de salud/ Hospitales área de consulta externa)

El Instituto Nacional de Salud Pública, está realizando un proyecto de investigación en colaboración con los Servicios de Salud en Chiapas. El objetivo del estudio es "realizar un diagnóstico de necesidades de salud sexual y reproductiva para la prevención del embarazo en la adolescencia en comunidades de Chiapas, además buscamos explorar e identificar la influencia de la epidemia por COVID-19 en la provisión de servicios para la prevención y atención del embarazo en la población adolescente". Estamos solicitando su participación a través del presente cuestionario auto-aplicado.
Este cuestionario está diseñado para ser llenado en el Centro de salud, servicio amigable o módulo para adolescentes, módulo de Planificación Familiar, medicina Preventiva o Consulta Externa

El cuestionario es anónimo

**Datos generales de la unidad médica**
Nombre de la Clínica/Hospital:<br>CLUES<br>[__|__|__|__|__|__|__|__|__|__|__|__]<br>______________________________________<br>Nombre del centro de salud/Clínica ______________________________<br>Localidad: _______________________________________
Fecha de aplicación del cuestionario: _______________<br>_______________________________

**Datos del entrevistado(a):**
Formación académica: ____

Tipo de plaza o categoría de la contratación:
___Base ____ Confianza ____Honorarios _____Servicio social _____ Interno _____Otro

Antigüedad en el cargo actual: años ______ meses_________

Edad (años cumplidos): _________

Sexo: Mujer_________ Hombre _____

A continuación, le voy a hacer unas preguntas relacionadas con la atención que ofrecen a los y las adolescentes en esta unidad de salud

**1. En esta unidad, ¿en qué turnos se ofrece atención a las y los adolescentes y cuántos días a la semana?**	En el turno matutino…………… 1<br>En el turno vespertino………… 2<br><br>No cuenta con este servicio…… 4 → **PASE a 4**<br><br>N°. de días<br>|__| |__| |__| **Puede seleccionar más de una opción**
**2. ¿Utilizan alguna guía, manual o protocolo para la atención de adolescentes en esta unidad?**	Sí……………………………….... 1<br>No…………………………………. 2
**3. ¿Cuál o cuáles?**	Acciones de línea de vida marcadas en una cartilla de salud para el adolescente o cartilla nacional de 10 a 19 años………………1<br>Guía de consejería en planificación familiar…………………..……2<br>Guía para la prevención y atención de infecciones por VIH y otras ITS……………………………………………………...........3<br>Guía didáctica o carta descriptiva de las acciones educativas……………………………………………………….....4<br>Marco jurídico sobre derechos sexuales y reproductivos de los adolescentes…………………………………………………………5<br>NOM-005-SSA2-1993 de los Servicios de Planificación Familiar...6<br>Criterios Operativos para los Servicios Amigables………………7<br>Programa de Salud Sexual y Reproductiva para Adolescentes.. 8<br>Procedimiento de planificación……………………………….……..9<br>Guía del enfoque integral para el trabajo con adolescentes...…10<br>Guía de Atención Médica y Manual de Consejería en Salud Reproductiva del Adolescente……………………………………..11<br>Guía para el manejo de grupos de adolescentes embarazadas………………………………………..……………..12<br>Otros _______________……………………………………………13<br>*(especificar)*

**4. ¿En esta unidad se cuenta con un espacio físico exclusivo y privado para brindar…**<br><br>***Puede seleccionar más de una opción***	**a)** las consultas de salud sexual y reproductiva para las y los adolescentes?................................|__| **b)** consejería sobre salud sexual y reproductiva para las y los adolescentes?.................................|__|	Opciones de respuesta:<br><br>Sí…………1<br>No………..2
**5. Seleccione los servicios de salud sexual y reproductiva que se ofrecen a las y los adolescentes en esta unidad médica**	Consejería u orientación sobre salud sexual y reproductiva……..... 1<br>Oferta de anticonceptivos……………………………………………… 2<br>Oferta de condones………..…………………………………………..….. 3<br>Oferta de anticonceptivos de emergencia………..……….………….. 4<br>Detección de ITS/infecciones o enfermedades de transmisión sexual………..……………………………………..……………… 5<br>Tratamiento para ITS/infecciones o enfermedades de transmisión sexual………..………………………………..……….……….. 6<br>Atención prenatal (sin especificar grupo de edad) …………………. 7<br>Atención prenatal específicamente diseñada para adolescentes…. 8<br>Consejería post evento obstétrico……………………………….…. 9<br>Grupos de apoyo o atención grupal para adolescentes embarazadas……………………………………………………………10<br>Actividades de educación sexual y reproductiva…………………… 11<br>Estrategias de comunicación educativa a través del internet (página de internet o redes sociales) ………………………….………..…. 12<br>Educación por pares………………………………………….……….. 13<br>Módulos ambulantes………………………………………….……….. 14<br>Creación de redes sociales de participación juvenil..……..……….. 15<br>Otro ____________________.......................…………………… 16<br>*(especifique)*	
**6. La consejería que se brinda a los adolescentes, ¿se ofrece de manera individual o grupal?**	Individual…………………………….... 1<br>Grupal………………………………… 2<br>Ambas (individual y grupal)………… 3<br>No se ofrece consejería……………. 4	
**7. Cuando brinda consejería a adolescentes, ¿de qué forma verifica que hayan comprendido sus indicaciones?**	Especificar:____<br>____<br>____	

A continuación, le voy a hacer unas preguntas relacionadas con la atención que usted ofrece a los y las adolescentes

**8. Antes del confinamiento por COVID-19, atendía <u>adolescentes</u> en esta unidad médica por…**	consejería de métodos anticonceptivos |__| solicitud de métodos……………………..…|__| anticonceptivos (citas de primera<br>vez y subsecuentes)?…………………………|__| embarazo?...|__| solicitud de vacunación contra el VPH?.........|__| Información de Infecciones de Transmisión Sexual<br>(ITS)/ VIH……………………………….……|__| solicitud de atención psicológica?.................|__| violencia familiar?...|__| violencia durante el noviazgo?.....................|__| abuso sexual?..|__| Otro______________________<br>especifique	Opciones de respuesta:<br>Sí…………1<br>No………..2
**9. Durante el confinamiento por COVID-19, atendió <u>adolescentes</u> en esta unidad médica por…**	embarazo?... |__| violencia familiar?... |__| violencia durante el noviazgo?......................... |__| abuso sexual?... |__| solicitud de vacunación contra el VPH?........... |__| información de Infecciones de Transmisión Sexual<br>(ITS)/ VIH………………………………………|__| consejería de métodos anticonceptivos solicitud de<br>métodos…………………………………….…|__| anticonceptivos (citas de primera vez y<br>subsecuentes)?... |__| solicitud de atención psicológica?.................... |__| <br>Otro______________________............................<br>(*especifique)*	Opciones de respuesta:<br>Sí…………1<br>No………..2
**10. ¿Existe algún caso en el cual NO otorgaría condones a un hombre o una mujer adolescente, aun cuando él o ella los solicite?**	Sí……………………………......... 1<br>No………………………………….. 2	Si el código es 2 pase a la pregunta 12.
**11. ¿En cuál o cuáles casos?**	Especificar:____<br>____<br>____	

**12. ¿Se realizan acciones concretas para integrar a los hombres adolescentes en la utilización de métodos anticonceptivos?**	Sí………………………………......... 1<br>No…………………………………….. 2	Si el código es 2 pase a la pregunta 14.
**13. ¿Cuáles acciones?**	Especificar:____<br>____<br>____	
**14. La consejería o promoción y orientación sobre el uso de métodos anticonceptivos, ¿se brinda a las(os) adolescentes menores de 18 años de edad que acuden sin acompañamiento de un adulto?**	Sí, a todos los menores de 18 años………….…0<br>Sí, Solo si son mayores de 15 años…….……..1<br>No, siempre deben estar acompañados de un adulto……………………………………………. 2<br>No aplica…………………………….…......... 3	
**15. ¿Se brindan métodos anticonceptivos a las(os) adolescentes menores de 18 años de edad que acuden sin acompañamiento de un adulto?**	Sí, a todos los menores de 18 años<br>Sí, Solo si son mayores de 15 años<br>No, siempre deben estar acompañados de un adulto………………………………….. 2<br>No aplica……………………......... 3	
**16 ¿Cuáles son los anticonceptivos más solicitados por los adolescentes?**	Condón ……………………………….……1<br>Pastillas de anticoncepción de emergencia o del día siguiente……………………………………2<br>Pastillas anticonceptivas………….…………… 3<br>Dispositivo, DIU o aparato………………………4<br>Implantes, tubos o norplant……………………..5<br>Parche anticonceptivo…………………………..6<br>Otro ____________________......................7<br>*(especifique)*	
**17. ¿Cuentan con archivos impresos o electrónicos de materiales de apoyo para la orientación y consejería para las y los adolescentes?**	Sí………………………………......... 1<br>No…………………………………….. 2<br>No sabe/No responde……………….. 9	
**18. ¿Se promueve la comunicación y la colaboración entre esta unidad y líderes comunitarios juveniles, grupos juveniles o escuelas locales?**	Sí………………………………......... 1<br>No…………………………………….. 2<br>No aplica……………………......... 3<br>No sabe/No responde……………….. 9	Si el código es 2, 3 o 9 pase a la pregunta 18.

**19. ¿Cómo se promueve?**	Especificar:___________________________________<br>____<br>____	
**20. ¿Qué hace cuando llega una usuaria a solicitar servicio y que sólo habla lengua indígena?**	Se pide apoyo al personal hablante de lengua indígena…………………. 1<br>Se solicita ayuda a la comunidad…. 2<br>No hay usuarias hablantes de lengua indígena……………………... 3<br>Otra (especificar)……………………. 4<br>No aplica…………………………... 5<br>No sabe/No responde……………… 9	Si el código es 3 pase a la pregunta 21.
**21. ¿Las pláticas de promoción y consejería anticonceptiva o de planificación familiar se brindan en lengua indígena a usuarios o usuarias adolescentes que lo requieran?**	Sí……………………………..........1<br>No…………………………………..2	
**22. ¿Cuentan con materiales en lengua indigena?**	Sí……………………………..........1<br>No…………………………………..2	
**23. ¿De qué métodos anticonceptivos se les *informa* a los hombres adolescentes?**<br><br>***Puede seleccionar más de una opción***	Condón o preservativo masculino….1<br>Condón o preservativo femenino…..2<br>Pastillas de anticoncepción de emergencia o del día siguiente…….3<br>Pastillas o píldoras hormonales……4<br>Inyecciones hormonales…………….5<br>Dispositivo o DIU………..………..6<br>Implantes (implanón o norplant)…..7<br>Parches………………………………8<br>Óvulos, jaleas, espuma o diafragma<br>Operación femenina, OTB o ligadura de trompas………………………….…9<br>Operación masculina o vasectomía.10<br>Ritmo, calendario, abstinencia periódica, termómetro, billings…….. 11<br>Retiro o coito interrumpido…………12<br>Otro (especificar)_______________ 13<br>Nada…………………………………..14<br>No sabe / No responde……………..99	
**24. ¿De qué métodos anticonceptivos se les *informa* a las mujeres adolescentes?**	Condón o preservativo masculino…1<br>Condón o preservativo femenino…..2<br>Pastillas de anticoncepción de emergencia o del día siguiente…….3<br>Pastillas o píldoras hormonales……4<br>Inyecciones hormonales…………….5<br>Dispositivo o DIU…..……..………6<br>Implantes o (implanón o norplant)..7<br>Parches………………………………8<br>Óvulos, jaleas, espuma o diafragma<br>Operación femenina, OTB o ligadura de trompas…………………9<br>Operación masculina o vasectomía.10<br>Ritmo, calendario, abstinencia	Seleccione todos los que la informante mencione.

	periódica, termómetro, billings……..11<br>Retiro o coito interrumpido…………12<br>Otro (especifique)……………………13<br>Nada…………………………………..14<br>No sabe / No responde……………..99	
**25.Además de la consejería, ¿qué otros mecanismos emplea para una mayor difusión e información de los métodos de planificación familiar?**<br><br>***ENTREVISTADOR (A): FAVOR DE NO LEER LAS OPCIONES DE RESPUESTA Reorganizar con las preguntas 26-28***	Difusión de material educativo impreso (folletos, trípticos, carteles, etc.)… …………………………………..1<br>Uso de material audiovisual……..……2<br>Difusión en televisión y/o radio local…………………………………...3<br>Uso de información disponible en internet<br>Educación por pares……………..……..4<br>Participación en ferias de salud sexual y reproducción.<br>/Módulos ambulantes……………….. 14<br><br>Sesiones educativas grupales en la unidad médica…………………………6<br>Participación en pláticas escolares /encuentros educativos o sesiones educativas grupales fuera de la unidad médica…………………………7<br>Periódico Mural………………………..8<br>Buzón (en la unidad médica) ………..9<br>Otros (especifique)…………….…….10<br>Ninguno……………………………..99	Seleccione todos los que la informante mencione.
**26.¿Para Ud. cuál cree que es su principal limitación para proporcionar información sobre salud sexual y reproductiva a adolescentes porque…**	tiene mucha carga de trabajo?....... |__| hay dificultades de espacio privado?...|__| hay pocas oportunidades de hablar solo(a) con usuarias(os) adolescentes (sin familiares o acompañantes)?........................... |__| hacen falta recursos materiales en la unidad médica?……….......... |__| hay diferentes creencias culturales y valores en la comunidad?……..... |__| no ha recibido suficiente capacitación?……………………….. |__| falta involucramiento de los hombres adolescentes? ..………… |__| falta involucramiento de los mujeres adolescentes?..…………………..… |__| falta involucramiento de la pareja sexual?……………………………..…. |__| **Otra _______________________ I__I**<br>**(*especifique*)**	Opciones de respuesta:<br><br>Si…………1<br>No………..2
**27. Mencione los tres métodos anticonceptivos que usted otorga con más frecuencia a hombres adolescentes.**	Condón o preservativo masculino…|__| Condón o preservativo femenino…..|__| Pastillas de anticoncepción de emergencia o del día siguiente…….|__|	

**Por favor, indique los métodos <u>iniciando de mayor a menor</u>.**	Pastillas o píldoras hormonales……|__| Inyecciones hormonales…………….|__| Dispositivo o DIU ……………………|__| Implantes (implanón o norplant)….. |__| Parches………………………………|__| Óvulos, jaleas, espuma o diafragma<br>Operación femenina, OTB o<br>ligadura de trompas…………………|__| Operación masculina o vasectomía.<br>Ritmo, calendario, abstinencia<br>periódica, termómetro, billings……..|__| Retiro o coito interrumpido………….|__| Otro (especificar)______________ |__| <br>Ninguno………………………………0<br>No sabe/No responde……………..9	
**28.Mencione los tres métodos anticonceptivos que usted otorga con más frecuencia a mujeres adolescentes.**<br><br>**Por favor, indique los métodos <u>iniciando de mayor a menor</u>.**	Condón o preservativo masculino…|__| Condón o preservativo femenino…..|__| Pastillas de anticoncepción de<br>emergencia o del día siguiente…….|__| Pastillas o píldoras hormonales……|__| Inyecciones hormonales…………….|__| Dispositivo o DIU ……………………|__| Implantes (implanón o norplant)….. |__| Parches………………………………|__| Óvulos, jaleas, espuma o diafragma<br>Operación femenina, OTB o<br>ligadura de trompas…………………|__| Operación masculina o vasectomía.<br>Ritmo, calendario, abstinencia<br>periódica, termómetro, billings……..|__| Retiro o coito interrumpido………….|__| Otro (especificar)______________ |__| <br>Ninguno………………………………0<br>No sabe/No responde……………..9	
**29. ¿Qué tan capacitado(a) se siente….**	**a.** para hablarles a los adolescentes sobre sus derechos sexuales y reproductivos?………………………|__| **b.** para brindar a los adolescentes consejería en salud sexual y reproductiva?……………..........|__| **c**. para hablarle a los adolescentes sobre las contraindicaciones y efectos colaterales de métodos de planificación familiar?……………|__| **d**. para insertar DIU?…………… |__| <br>**g.** prescribir anticoncepción de emergencia?…………………….. |__| **h**. en temas de igualdad de género?………………………… |__|	Códigos de respuesta:<br><br>No capacitado……0<br>Poco capacitado…1<br>Medianamente capacitado………..2<br>Suficientemente capacitado………3

**30. Durante los últimos cinco años, ¿recibió capacitación en…**	**a**. Derechos sexuales y reproductivos de los adolescentes? …………………………….…. |__| **b**. Consejería en salud sexual y reproductiva……………………………….…. |__| **c**. Anticoncepción para adolescentes…….…. |__| **e**. Anticoncepción de emergencia………….. |__| **f.** Temas de igualdad de género……….…… |__| **g.** Actualización en anticoncepción postevento obstétrico?... …… |__| <br>Otro________________________<br>especifique<br><br>NO ha recibido capacitación	Opciones de respuesta:<br><br>Sí………..1<br><br>No……….2
31. ¿Cuenta con internet (casa o en la unidad médica)?	Sí, en casa……………………………1<br>Sí en la unidad médica……………..2<br>No cuento con internet……………...0	
32. ¿Le interesaría capacitarse por medio de un curso virtual?	Sí……………………………………1<br>No……………………………………2	
33. ¿Considera que cuenta con el apoyo institucional para realizar una capacitación en el trabajo?	Sí……………………………………1<br>No……………………………………2	
